# The Future of Dental Implants: A Narrative Review of Trends, Technologies, and Patient Considerations

**DOI:** 10.7759/cureus.90380

**Published:** 2025-08-18

**Authors:** Johnson Raja James, Avina Kharat, Suresh Chinnakutti, Snehal Kamble, Mita Mandal, Anubhav Das

**Affiliations:** 1 Department of Periodontics and Implantology, Rajas Dental College and Hospital, Tirunelveli, IND; 2 Department of Pharmacology, Employees' State Insurance Corporation (ESIC) Medical College and Hospital, Indore, IND; 3 Department of Oral and Maxillofacial Surgery, Vinayaka Missions Sankarachariyar Dental College, Vinayaka Missions Research Foundation (Deemed to be University), Salem, IND; 4 Department of Obstetrics and Gynecology, Bhausaheb Mulak Ayurved Mahavidyalaya, Nagpur, IND; 5 Department of Orthodontics and Dentofacial Orthopedics, Guru Nanak Institute of Dental Sciences and Research, Kolkata, IND; 6 Department of Oral and Maxillofacial Surgery, Rajiv Gandhi University of Health Sciences, Bengaluru, IND

**Keywords:** 3d printing, artificial intelligence, biocompatibility, implantology, osseointegration

## Abstract

This narrative review examines recent advancements in dental implantology, emphasizing innovative materials, digital technologies, and patient-centered approaches that improve treatment outcomes. Implant systems have progressed from traditional titanium to biocompatible alternatives such as zirconia, titanium-zirconium alloys, and scaffold-based designs, enabling enhanced integration and durability. The review draws on literature published between 2015 and 2025, identified through structured searches of biomedical databases including PubMed, Scopus, and Google Scholar. Developments in artificial intelligence, robotics, and 3D printing have enhanced surgical precision and planning accuracy; however, their clinical adoption remains limited by high costs, steep learning curves, and inconsistent long-term data. While bioactive surfaces, regenerative biomolecules, and minimally invasive techniques contribute to better healing outcomes, their accessibility and regulatory approval vary widely across regions. Smart implants with sensor technologies show promise for real-time monitoring but lack robust clinical validation and standardization. Ethical concerns surrounding artificial intelligence (AI) integration, patient data privacy, and equitable access further complicate implementation. Incorporating sustainable practices and personalized treatment strategies may enhance the predictability and success of implant procedures, but these must be weighed against economic and logistical constraints. These considerations underscore the need for critical evaluation as implant dentistry undergoes a paradigm shift in modern oral rehabilitation.

## Introduction and background

This narrative review discusses the development and recent progress in dental implantology based on peer-reviewed literature between 2015 and 2025. The emphasis is on material development, digital technology, and patient-focused practices that enhance treatment outcomes, longevity, and functionality. Dental implants play a crucial role in contemporary dentistry for tooth replacement owing to their greater hardness, aesthetic integration, and functional efficiency compared to conventional prosthetics [1]. Their extensive clinical success can fundamentally be attributed to the process of osseointegration, where materials like titanium and zirconia that are biocompatible directly integrate with bone tissue, providing long-term structural stability and great survival rates [2].

The theoretical ground upon which tooth replacement is based, however, is not new. Archaeological research indicates that ancient civilizations, such as the Mayans and Egyptians, employed rudimentary dental implantation techniques using carved seashells, ivory, and gold [3,4]. Although these techniques were not based on science, documentation of some degree of bone integration indicates an enduring human curiosity about the replacement of permanent teeth. The contemporary implant era started with Per-Ingvar Brånemark's seminal finding in 1952 that titanium formed a direct and stable bond with bone [5]. His accomplishment in 1965 of placing a titanium implant, which was functional for more than four decades, was a turning point in clinical dentistry and resulted in the guiding principles of modern implant practice [6].

The intervening years have seen advances in technology transform the discipline. Computer-aided design and manufacturing (CAD/CAM), cone-beam computed tomography (CBCT), and 3D printing have dramatically enhanced diagnostic precision and implant planning [7]. These technologies allow clinicians to design patient-specific surgical guides that improve accuracy, reduce operating time, and decrease postoperative complications [8]. Artificial intelligence (AI) and robotics have further improved procedural precision, providing real-time feedback during surgery and adaptive control [9]. Widespread adoption is, however, moderated by considerations of high expense, training requirements, and the necessity of long-term validation over diverse patient populations [10].

Material development is also at the forefront of promoting implant success. Titanium is still the gold standard because of its excellent mechanical and biological properties [11]. Zirconia, however, has picked up steam as an alternative in metal sensitivity or aesthetic preference cases [12]. Short- to mid-term results are encouraging but are still in need of research to demonstrate long-term clinical equivalency with titanium [13]. Despite these developments, implant dentistry continues to be plagued by chronic biological issues, especially peri-implantitis, a bacterial infection that adversely affects implant success [14]. The estimated prevalence varies between 10% to 50%, depending on factors related to systemic health, smoking, and oral hygiene [15]. Advances in antimicrobial coatings, bioactive surfaces, and regenerative methods such as stem cell therapy and growth factors have the potential to counteract these risks, although evidence-based clinical protocols are yet to be developed [16,17].

Incipient smart implants with biosensors have the potential for real-time assessment of mechanical load, implant stability, and tissue well-being [18]. Combined with AI-driven diagnostics, they can potentially facilitate individualized and predictive treatment paradigms [19]. These innovations are still in nascent development stages and raise ethical and regulatory questions about data privacy, equity, and clinical validity [20]. This review provides a balanced and critical assessment of implant dentistry's historical background, existing developments, and future directions. It also mentions existing limitations, controversies, and gaps in research required for safe, effective, and affordable innovation within the discipline.

## Review

This section provides a narrative review of novel developments in dental implant materials and constructs that have been organized in a critical approach that reviews the innovation in biocompatibility, osseointegration, and the smart faculty. Peer literature was considered using relevant searches in databases, like PubMed, Scopus, and Google Scholar, that were searched specifically throughout 2015-2025. The selection method was PICO-style framed to pursue: adult patients and the necessity to receive dental implants (Population), the application of innovative implant materials and technologies (Intervention), the comparison with the conventional titanium-based implants (Comparison), and the outcomes regarding its clinical results, which include osseointegration, long-term stability, and the complication rates (Outcomes). All experimental studies, clinical trials, in vitro studies, and review articles were identified to provide a systematic review of well-known and new evidence in the field of implantology. A preference was observed when the study product reported clinical relevance, translational viability, and performance, with its outcome to be measured in various aspects.

Advances in dental implant materials and design

Evolution of Implant Materials

The development of new dental implant materials established implantology through advances in stability, together with excellent compatibility and improved aesthetics. The adoption of titanium implants, specifically commercially pure titanium (CpTi) and titanium-6%-6% aluminum-4%-4% vanadium (Ti-6Al-4V) alloy, became widespread because of their excellent combination of biocompatible characteristics, durable performance, and superior mechanical strength. Medical researchers pursued new materials because of worries about soft-tissue color changes, metal corrosion, and sensitivity reactions [18]. Zirconia emerged as a promising metal-free substitute, offering aesthetic benefits, corrosion resistance, and favorable tissue integration. Preclinical and early clinical studies have demonstrated that zirconia implants with modified surfaces can achieve bone-to-implant contact levels and osseointegration outcomes comparable to titanium [19]. Surface modifications, including sandblasting combined with acid etching and laser treatments, enabled zirconia implants to achieve similar bone-implant contact and stability rates to titanium implants. However, zirconia remains more brittle under occlusal forces, which limits its use in posterior or high-load areas, and long-term multicenter trials remain limited.

The creation of titanium-zirconium (Ti-Zr) alloys provided dentists with strong and fatigue-resistant dental implants that contained zirconia and titanium to create narrower implant structures [20]. Recent in vivo evidence supports their enhanced mechanical strength and osseointegration capacity, especially for narrow-diameter implants. Nonetheless, Ti-Zr systems lack extensive long-term data beyond five years, and their performance in patients with compromised bone density remains under investigation.

The medical field has developed root-analogue implants (RAIs) as a new design that duplicates the natural tooth root structure to deliver a better fit while minimizing surgical complications. Initial experimental applications of zirconia and titanium-based RAIs have shown promising osseointegration and aesthetic results in small case series and pilot models [21]. Zirconia and titanium-based RAIs perform effectively for osseointegration and aesthetic outcomes, which minimizes the necessity of extensive modifications when placing them. However, limited standardization and high customization demands restrict their widespread clinical adoption.

**Figure 1:**
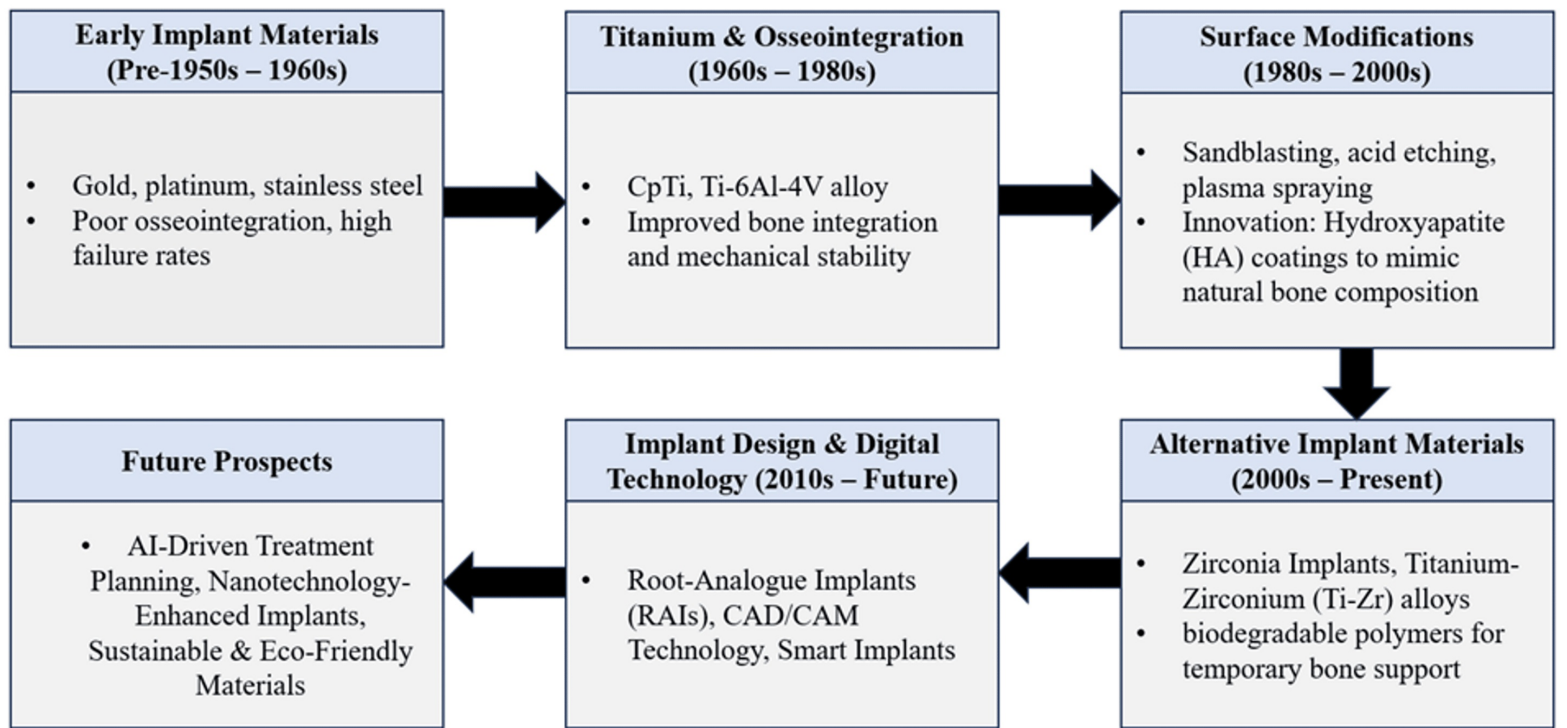
Evolution of dental implant materials and design Image Credit: Johnson Raja James CpTi: commercially pure titanium; Ti-6Al-4V: titanium-6%-6% aluminum-4%–4% vanadium alloy; HA: hydroxyapatite; Ti-Zr: titanium-zirconium; RAIs: root-analogue implants; CAD/CAM: computer-aided design/computer-aided manufacturing; AI: artificial intelligence

Present-day implantology receives advanced precision from CAD/CAM systems, which produce personalized implants and prosthetics according to patients' anatomical needs. The use of CAD/CAM technology brings revolutionary changes to implant planning and fabrication because it delivers better precision with enhanced mechanical interlocking and superior long-term stability [22]. Evidence from clinical applications shows that CAD/CAM-assisted fabrication improves prosthetic fit and reduces treatment time. However, integration requires high-cost digital infrastructure and trained personnel, which can limit accessibility in low-resource settings. The evolution of dental implants and the materials used in them are depicted in Figure 1.

Biocompatibility and Osseointegration Advancements

Biocompatibility combined with osseointegration function is a crucial requirement to obtain enduring dental implant achievements. The materials used in implants need to stop negative biological responses and simultaneously encourage the direct bonding between bone tissue and implants. Advances in material science and surface modifications have significantly improved implant success rates and treatment predictability [22]. Studies confirm that roughened or coated implant surfaces improve early healing and long-term integration.

Titanium continues to set the standard in dentistry because it integrates properly with bones and maintains high structural stability, which produces outstanding success rates. The material establishes powerful bone bonding, which lowers the possibility of tissue rejection. The development of zirconia (ZrO₂) implants, Ti-Zr alloys, hydroxyapatite (HA)-coated implants, and bioactive glass occurred because of concerns about metal allergies and peri-implantitis and limitations regarding aesthetics [22,23]. However, titanium may still trigger peri-implant inflammation in susceptible patients.

Medical science uses zirconia implants to provide patients with metal-free implants that both adapt well to the body and duplicate natural tooth appearance. Surface-modified zirconia implants have demonstrated osseointegration levels similar to titanium in small clinical studies [24], but they maintain brittleness when subjected to occlusal forces. The combination of titanium strength and zirconia compatibility in Ti-Zr alloys enables better fatigue resistance, especially when used for narrow-diameter implants [25]. Yet, large-scale clinical evidence remains limited.

The biomimetic characteristics of HA-coated implants enhance bone growth, while bioactive glass demonstrates anti-inflammatory and regenerative potential [26]. Both materials show promise but require further standardization for consistent long-term outcomes. The biocompatibility and osseointegration advancements in dental implant materials are mentioned in Table 1.

**Table 1:** Biocompatibility and osseointegration advancements in dental implant materials Ti: titanium; ZrO_2_: zirconia; Ti-Zr: titanium-zirconium; HA: hydroxyapatite; PEEK: polyetheretherketone

Material	Biocompatibility	Osseointegration	Surface Modification	Clinical Advantages	Challenges
Ti	Excellent, well-documented	Highly predictable	Sandblasting, acid etching	Proven long-term success	Risk of peri-implantitis
ZrO_2_	High, non-metallic, reduced inflammation	Comparable to titanium with modifications	Laser treatment, surface roughening	Aesthetic benefits, metal-free	Brittle under stress
Ti-Zr alloy	Improved over pure titanium	Superior mechanical properties	Dual-phase alloy treatment	Stronger, thinner implants are possible	Limited long-term data
HA-coated implants	Enhanced due to HA-coating	Enhanced bone integration	Plasma spraying	Stimulates bone growth, higher integration	Coating degradation over time
Bioactive glass	Promotes bone regeneration	Bioactive properties support osseointegration	Chemical grafting	Reduces inflammation, bioactive response	Expensive, clinical validation needed
PEEK	Moderate, improved with coatings	Moderate without modifications	Surface functionalization	Flexible alternative	Lower osseointegration without surface modification
Graphene-based implants	High, stimulate cell growth	Enhances osseointegration via bioactivity	Coating with osteoconductive molecules	Potential for future applications	Experimental stage, high cost
Nanostructured surfaces	Improved cellular interaction	Nanotopography improves integration	Nanotexturing, coatings	Better early-stage healing	Complex manufacturing
Stem cell-enhanced implants	Supports osteogenic differentiation	Accelerated healing potential	Bioactive factor loading	Regenerative potential	Requires further research

3D-Printed and Bioengineered Implants

Bioengineering combined with 3D printing technologies has changed dental implantology because it enables customized implants, better tissue integration, and improved long-term reliability. 3D-printed and bioengineered implants address traditional weaknesses by delivering tailored solutions that boost clinical results [27]. The additive manufacturing technology used in 3D-printed dental implants enables precision production that matches human anatomy. Medical professionals use CBCT and intraoral scanning to create digital models for guiding the 3D printing of titanium, zirconia, and polymer-based implants [28]. Studies have shown that porous 3D-printed titanium implants improve osseointegration by mimicking trabecular bone [29]. Dental professionals use individualized implant designs to reduce surgical invasiveness, cutting surgery duration and recovery time [30]. However, clinical validation beyond small case series remains limited, and production cost is a key constraint.

Customized surgical guides and prosthetics are major 3D printing applications, helping improve alignment and reduce failure risk. Evidence supports improved placement accuracy with these tools, especially in complex anatomical regions. Studies show that 3D-printed zirconia implants with hydroxyapatite coatings demonstrate superior biomechanical properties and faster integration [31]. Still, zirconia’s brittleness under occlusal stress limits its application in high-load areas.

Tissue engineering techniques support the creation of implants that integrate with biological tissue. Stem cell-based scaffolds have shown success in generating vascularized bone in animal models. Scientists have created functional tooth buds capable of forming neural and vascular structures. Research indicates that bioengineered tooth buds placed in alveolar bone support structural and functional development [32]. These remain in the experimental stage, with ethical and clinical barriers preventing human application.

Bioactive coatings enhance healing and integration. Bioengineered implants benefit from growth factor-enriched hydrogels and peptide-functionalized surfaces that promote cell attachment and bone regeneration while reducing failure risk [33]. In addition to peptides, coatings incorporating silver nanoparticles, calcium phosphate, bioactive glass, and antibiotics have been developed to improve antibacterial performance and tissue compatibility. Preclinical evidence supports early-stage healing, but consistent performance and delivery control are ongoing challenges. Silver nanoparticles exhibit broad-spectrum antimicrobial effects, though concerns persist regarding cytotoxicity and resistance development [11]. Calcium phosphate and hydroxyapatite coatings stimulate osteoblast adhesion and early bone bonding, yet may degrade unpredictably over time [20]. Bioactive glass promotes tissue regeneration and modulates inflammation, though cost and mechanical limitations reduce its widespread use [6]. Graphene coatings demonstrate both antimicrobial activity and improved bone bonding, helping to reduce peri-implantitis [16]. Despite positive in vivo results, long-term clinical data and regulatory approval are still pending. Overall, while bioactive coatings offer targeted functional enhancements, their long-term effectiveness, reproducibility, and clinical validation remain key barriers to integration into routine implantology.

Bio-hybrid implants combine engineered periodontal ligament tissues with implant structures to restore natural attachment and proprioception. This leads to better chewing efficiency and long-term stability [34]. However, these are still in the developmental phase, with scalability and clinical translation yet to be achieved. The combination of 3D printing and bioengineering offers biomimetic implant solutions with promising functional outcomes.

Smart Implant Materials With Enhanced Properties

Modern dental implant technology continues to focus on material advancement for better biocompatibility, along with osseointegration capacity, while adding antibacterial properties for real-time monitoring to advance long-term functionality rates and lower failure rates. The combination of biosensors with bioactive coatings, along with nanotechnology applications, delivers maximum performance enhancement for implants situated in the oral cavity [35].

Titanium implants achieve widespread clinical use because they display important properties, including resistance to corrosion, strong bone integration, and excellent biocompatibility. Bioactive coatings combined with nanostructures strengthen the implant-bone bonding and simultaneously minimize bacterial settlement on the surface. Current titanium implant designs come with biosensors that track implant stability while identifying signs of implant failure and peri-implantitis very early [36].

Studies reveal that zirconia implants stand apart because they contain no metal substances while simultaneously providing excellent tissue acceptance and enduring against corrosion. The ability of zirconia to bond with bone tissues substantially improves due to laser modifications while simultaneously enhancing its material strength [37]. Surface modifications by applying graphene coatings produce stronger and more conductive medical implants. Medical researchers have determined that graphene coatings enhance bone regeneration along with improved attachment between tissue and accelerated healing, during which they simultaneously impede bacterial infections and biofilm development [38]. Implants with antimicrobial coatings that contain silver nanoparticles, together with peptide coatings and bioactive glass, fight infections while improving bone growth to enhance implant stability as well as resist infections [39].

**Figure 2:**
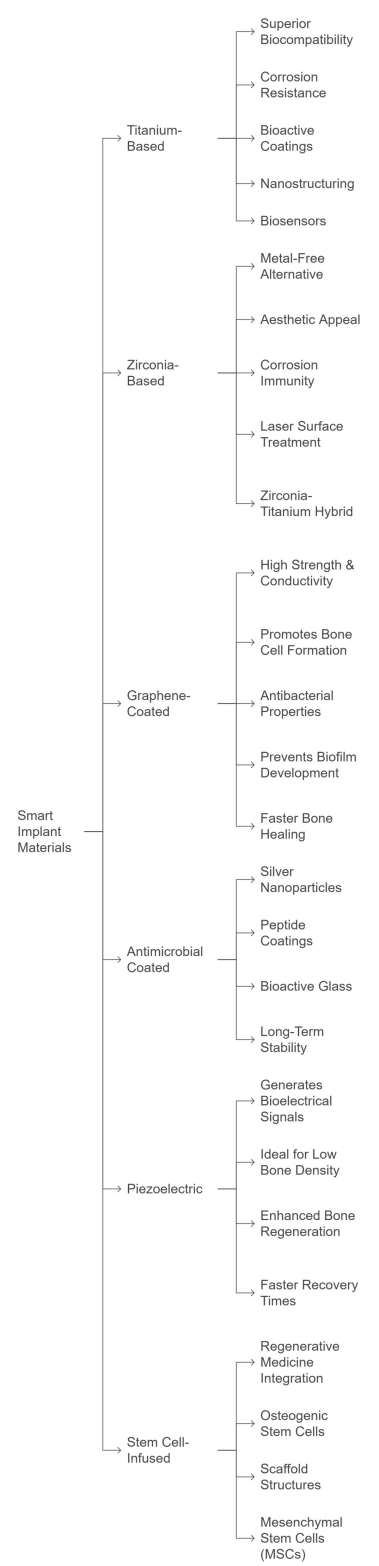
Mindmap of smart implant materials with enhanced properties in modern dental implantology Image Credit: Johnson Raja James MSCs: mesenchymal stem cells

Implants with piezoelectric properties produce electrical signals through mechanical force to promote bone growth, which speeds up the healing process of patients with low bone density and strengthens implant success rates. Surgical implants enriched with stem cells, paired with bioengineered structures combined with osteogenic stem cells, facilitate quick bone healing while they improve the surgical implant connection to host tissue [40]. The advancements in smart implant materials and the innovations in dental implantology are depicted in Figure 2.

Innovations in implantology techniques and procedures

Innovations in implantology techniques and procedures have significantly enhanced implant planning, placement accuracy, and restoration efficiency. Treatment speeds have increased while surgical trauma decreased, and osseointegration improved through digital workflows, AI-driven planning, robotic assistance, immediate loading protocols, and minimally invasive surgical approaches [41]. Multiple clinical studies have confirmed improvements in procedural efficiency, healing time, and patient-reported outcomes with these technologies, especially when implemented in structured, digitally guided workflows [25,32].

Digitally assisted implant surgery, using CBCT, intraoral scanners, and 3D-printed surgical guides, enables precise implant placement and reduces surgical trauma [10]. These technologies have demonstrated improved placement accuracy and reduced intraoperative errors in controlled clinical settings [18]. The implementation of AI-based planning software and robotic-assisted systems, which process CBCT data to suggest optimal implant positions, represents a shift toward semi-automated precision surgery. Robotic surgery with haptic feedback provides real-time control and improved stability, leading to enhanced surgical outcomes [42]. However, robotic systems remain cost-intensive, with limited global accessibility and few multicenter trials assessing long-term clinical superiority.

The immediate loading technique, allowing prosthetic placement directly after implant surgery, yields favorable functional and aesthetic outcomes, particularly for fully edentulous patients. Surface-treated implants and optimized macrodesigns contribute to primary stability, which is critical to success [43]. Although short-term studies support its effectiveness, success depends on stringent patient selection and surgical control, with early failure risks in poorly managed cases [36]. Minimally invasive methods, such as flapless surgery, reduce postoperative discomfort and healing time. Guided implant placement using digital templates ensures high placement accuracy and reduces complications [44]. Despite their validation in clinical trials, adoption of these protocols is still uneven due to equipment costs, operator training needs, and workflow integration barriers. The innovation techniques and procedures followed in dental implantology are mentioned briefly in Table 2.

**Table 2:** Innovations in implantology techniques and procedures

Technique/Procedure	Functionality	Accuracy Improvement	Clinical Benefits	Osseointegration Impact	Time Efficiency	Patient Comfort	Limitations	Future Potential
Digital dentistry and guided surgery	Enhances precision and reduces errors	Real-time navigation for implant placement	Reduces the risk of implant failure	Accurate bone assessment improves integration	Reduces surgical and planning time	Minimally invasive and more predictable	High cost and training requirements	Integration with AI for real-time feedback
Computer-aided design and manufacturing (CAD/CAM)	Designs and fabricates precise implant prosthetics	Digitally designed for a perfect fit	Enhances prosthetic longevity	Precise load distribution for stability	Speeds up prosthetic fabrication	Reduces the need for trial fittings	Requires a specialized digital workflow	Further advancements in digital workflows
Robotics and AI in implantology	AI-assisted planning and robotic surgery	Predictive analytics optimize implant positioning	Optimizes surgical workflow	Improves bone response to implants	Automates the implant positioning process	Less human error, improved safety	Dependence on AI reliability	Increased automation in implantology
Immediate loading techniques	Enables immediate placement of a prosthesis	Ensures primary stability for early loading	Faster restoration and functional recovery	Stimulates bone growth under early load	Shortens treatment duration	Immediate function with minimal wait time	Requires strict case selection	Broader application in edentulous cases
Minimally invasive surgery	Reduces surgical trauma and healing time	Reduces damage to surrounding tissues	Minimizes patient discomfort	Less disruption to the osseointegration process	Minimizes intraoperative time	Smaller incisions, less post-op pain	Potential limitations in complex cases	More refined techniques for soft tissue preservation
3D-printed customized implants	Custom implants tailored to patient anatomy	Improves adaptation and osseointegration	Enhances long-term success rates	Custom-designed implants optimize stability	Faster fabrication of patient-specific implants	Eliminates the need for excessive modifications	Expensive technology with limited accessibility	Improved affordability and accessibility

Role of biotechnology and regenerative approaches

Biotechnology and regenerative science have taken the field of implantology to a new level by allowing the advanced regeneration of tissue, the development of individualized implant plans, and shortened healing time. These innovations span clinically available, preclinical, and experimental stages. Technologies that are available clinically are bioactive coatings that stimulate osseointegration and minimize the risk of infection [45]. The use of AI in treatment planning and 3D printing of customized implants has been applied more frequently in clinical practice and provides greater precision and customization [46].

Early-phase clinical and preclinical technologies involve the application of mesenchymal stem cells (MSCs) (derived via bone marrow, adipose tissue, or dental pulp), which have shown osteogenic, angiogenic, and immunomodulatory properties in animal-based studies and small-scale human trials [32]. Likewise, other growth factors like BMP-2, BMP-7, VEGF, and PDGF have demonstrated promise in improving bone and soft tissue regeneration during pilot studies, but their frequent application remains constrained by regulatory inhibitions following issues of uncontrolled tissue growth and limited delivery localization [47].

Experimental/investigational technologies include genomic screening to generate implants that are personalized implants, designing implants guided by genes, and modifying host response based on gene-based applications, which are in early stages of research with no clinical use at present [48]. Real-time monitoring of integrated biosensors of peri-implant tissue health is also actively developed, and there is some availability in pilot settings [30].

Such emerging treatments, especially those that deal with stem cells and gene editing, have many regulatory and ethical issues. The limitations are standardization of tissue sourcing, ethical informed consent, long-term patient follow-up, and tumorigenesis or genetic drift risks. Moreover, the inequality in access, including the cost of access and the absence of regulatory infrastructure in low-resource contexts, increases the issues of equity of clinical adoption. Existing regulatory authorities, such as the FDA and European Medicines Agency (EMA) have not issued universal guidelines on the use of these advanced therapies in the dental field, indicating the necessity of strong frameworks, open reporting, and global partnership to achieve safe, ethical, and inclusive translation into implant dentistry [11]. Patient-centered therapies and patient-specific implant solutions are mentioned in Table 3.

**Table 3:** Personalized medicine and patient-specific implant solutions CAD/CAM: computer-aided design and manufacturing

Aspect	Functionality	Customization Level	Clinical Benefits	Technology Used	Time Efficiency	Patient Comfort	Limitations	Future Potential
Genetic profiling for implant success	Identifies genetic predispositions affecting osseointegration	Patient-specific genetic markers guide treatment	Improves implant success rates by minimizing risk factors	Genomic sequencing and biomarker analysis	Speeds up preoperative planning and risk assessment	Minimizes the risk of implant failure due to genetic mismatches	High cost and need for specialized testing	Widespread integration of genetic screening in implantology
3D-printed patient-specific implants	Creates implants tailored to patient-specific anatomy	Fully customized implant shape and size	Reduces complications and enhances long-term stability	3D printing and CAD/CAM technology	Reduces surgery time with a precise fit	Ensures better fit and function	Limited access to patient-specific 3D printing facilities	Expansion of 3D printing for mainstream clinical use
Customized surface modifications	Enhances osseointegration based on patient bone response	Tailored surface coatings for optimized healing	Accelerates bone healing and integration	Nanocoating, plasma spraying, and bioactive layers	Decreases healing time through enhanced integration	Reduces inflammation and enhances healing	Complexity in determining the ideal surface treatment	Development of bioactive coatings with real-time adaptability
AI-driven treatment planning	Uses AI to optimize implant positioning and material selection	Personalized implant planning using digital models	Ensures high accuracy in implant placement	Machine learning and AI-based diagnostics	Shortens diagnosis-to-surgery timeframe	Optimizes treatment with reduced chairside adjustments	Potential errors in AI-driven decision-making	Advanced AI models with precision diagnostics
Stem cell-based regenerative implants	Incorporates stem cells for patient-specific regeneration	Adapted to individual regenerative potential	Faster healing and improved tissue compatibility	Stem cell engineering and bioprinting	Speeds up tissue regeneration post-surgery	Less post-operative discomfort and faster recovery	Ethical and regulatory challenges with stem cell use	Refinement of stem cell applications in oral regeneration
Personalized drug-eluting implants	Delivers localized medication to prevent infection and inflammation	Controlled drug release based on patient needs	Prevents peri-implantitis and other complications	Nano-drug carriers and biodegradable coatings	Provides long-term localized drug therapy	Eliminates the need for systemic medication post-surgery	Uncertainty in the long-term effectiveness of drug-eluting coatings	Smart implants with controlled and responsive drug delivery

Patient-centered considerations and outcomes

All of the patient-related factors should be considered in terms of general systemic health, behavioral habits, psychological well-being, and economic conditions to ensure the success and long-term survival of dental implants. Other conditions, like diabetes, osteoporosis, and autoimmune diseases, have an adverse impact on osseointegration by worsening bone remodeling, decreasing vascularization, and destabilizing immune balance [49]. Hyperglycemia negatively impacts bone metabolism and microvascular response in uncontrolled diabetes, which further results in poor implant stability and a higher likelihood of peri-implant complications [50]. In the same manner, by reducing bone density and turnover, osteoporosis interferes with the anchorage of implants, especially in the maxilla [18].

Smoking also exacerbates bone healing by decreasing blood flow in capillaries, inhibiting fibroblast activity, and encouraging microbial implantation, all of which increase the chances of implant failure [51]. Patients on bisphosphonates, particularly intravenous ones, are at a higher risk of medication-related osteonecrosis of the jaw (MRONJ), and they require modified treatment plans [26]. In the course of time, the success of implants is dictated by both biological and mechanical aspects. The survival rates are 90-95% at 10 years according to clinical data, yet complications are still present, including peri-implantitis, mechanical fractures, and prosthetic failures [52]. The prevalence of peri-implant diseases in patients with implants reaches 28-30%, and the causes are frequently improper oral hygiene, biofilms, and inflammatory reactions of the host [14]. The mechanical problems are connected with loosening of screws, fractures caused by bruxism, and prosthetic malfitting, which is often related to occlusal overload [10]. It has been proven that professional maintenance programs, follow-ups, and patient education contribute to a significant decrease in complications and increase the implant lifespan [53].

In addition to clinical measures, patient-reported outcomes are crucial to the assessment of implant success. Happiness is directly associated with comfort, functional performance, and beauty, and speech and social confidence enhancements [28]. The digital design of smiles and the customization of prosthetic pieces prove to be more precise in aesthetics and more accepted by patients [43]. Such strategies lead to psychological well-being, and research has shown that self-esteem and social reintegration are enhanced after treatment [54]. Nonetheless, cost-related issues are a significant impediment to the availability of implants. The cost of treatment, exclusion of implants by insurance, and adjunctive procedures like sinus augmentation tend to discourage patients who might be interested in implant therapy [35]. In turn, patients with limited resources prefer cost-effective options such as mini-implants or implant-retained overdentures [46]. Although the upfront cost of implant therapy is expensive, it has been demonstrated to be cost-effective in the long run due to fewer maintenance requirements and improved quality of life [15].

Current limitations and translational gaps in implant innovation

Although there has been stunning advancement in both the materials and design of implants, several barriers still curtail the regular clinical use of the emerging technologies. Those innovations include the use of stem cell-incorporated scaffolds, bioactive surfaces, piezoelectric lamina, and bioengineered periodontal ligamentous structures, which are promising in laboratory research and preclinical trials. Nonetheless, there is still a lack of well-conducted clinical trials confirming their safety, efficacy, and longevity. Other options that have greater biological promise are technologies such as graphene-coated and drug-eluting implants, but they suffer limitations in terms of cost, complicated processes, a need to be standardized, and not being approved by regulators. On the same line, the brittleness of zirconia and rather short-term data of Ti-Zr alloys limit wider application in high-load areas. Although 3D-printed patient-specific implants are anatomically precise, their manufacturing cost, low reimbursement system, and vague superiority vis-à-vis standard implants restrain their wide use.

Such translational gaps are not only scientific but also structural. Individual differences in trial design, the non-standard reporting of outcomes, and a lack of agreement on clinical anticipated standards serve to deter comparative analysis and integration of guidelines. More logistical and ethical issues, including the question of where to obtain stem cells, how to provide access to genetic screening, and the understandability of the decisions made by AI tools, continue to slow acceptance. Most innovations do not have post-market surveillance and real-world validation, particularly in different populations. This has led to a situation where most patients cannot access good technologies, which further exposes them to disparities in advanced dental care. It will take an interdisciplinary approach, harmonization of regulations, and fair policy frameworks to overcome these gaps. And it is only in this way that avant-garde advances can be brought from bench to chairside so as to enhance outcomes with safety, affordability, and accessibility.

Future directions and challenges

The field of implant dentistry is fast developing, with the use of new biotechnologies, AI, and sustainability-oriented research, all designed to enhance the life of implants, clinical performance, and patient-centered treatment. Technologies that are clinically implemented, like digital workflows, computer-aided implant planning, and 3D printing, have increased surgical accuracy and eliminated human error in regular practice [55]. Implant planning and risk assessment with predictive analytics and AI-based decision support systems are increasingly used, with results showing increases in procedural efficiency [55]. During investigation, researchers are engineering bioengineered implants, which are a combination of MSCs and growth factors along with tissue scaffolds to facilitate bone regeneration and osseointegration [56]. In a similar manner, the development of biosensor-implanted devices is in progress to enable the constant tracking of occlusal forces and the early diagnosis of peri-implant complications [56]. These technologies are mostly in preclinical or early clinical trials, and they have not yet been commonly integrated into standard care, although they are promising.

Others, including the use of nanostructured coatings or a combination of gene therapy and implant design, are less viable, more theoretical, or in their very early experimental stages. Although there is evidence that such technologies might increase the compatibility of soft tissues or minimize the formation of biofilms, their clinical performance has not been confirmed in long-term studies [56]. Despite the innovation, significant barriers remain. Custom-designed implants, regenerative materials, and AI infrastructure are expensive, and hence, they are not accessible, especially to underserved populations [56]. Emergent procedures are not typically covered by insurance, and regulatory bodies, including the FDA or EMA, have lengthy approval processes regarding AI diagnostics, 3D-printed systems, and stem cell therapies [56]. These regulatory challenges are compounded by ethical considerations. As an example, the application of AI in treatment decision-making is associated with the risk of data privacy, bias in algorithms, and loss of autonomy in patients [57]. There are also concerns about gene-based and stem cell therapy regarding informed consent, moral acceptability, and health equity in access to innovative therapies [57]. There must also be international initiatives to come up with transparent, standardized, and fair regulatory guidelines that will govern these tools.

Meanwhile, the dental implant industry is becoming more sustainability-minded. Scientists are working on biodegradable materials and recyclable packaging, and eco-efficient manufacturing methods to minimize the environmental footprint [58]. Manufacture of titanium and zirconia implants is already associated with significant energy-intensive raw material extraction and production, but low-energy additive manufacturing and nanostructured surface modifications are under development to minimize resource use [58]. The incorporation of green dentistry concepts such as digital workflow and low-waste procedures has placed the discipline in a better position to be more environmentally friendly [58]. In the future, a synergetic equilibrium between advancement in technology, patient safety, regulatory control, and environmental sustainability will be necessary to achieve successful implant innovation.

## Conclusions

Advancements in dental implantology driven by materials science, digital technology, and regenerative medicine have significantly improved implant success and longevity. The shift from conventional titanium implants to newer materials like zirconia, Ti-Zr alloys, and bioengineered scaffolds has enhanced biocompatibility, osseointegration, and aesthetics. Clinically adopted tools such as AI-guided planning, robotics, and 3D printing have improved surgical accuracy and reduced complications. Minimally invasive techniques, including flapless placement and immediate loading, have increased patient comfort and shortened recovery. In contrast, emerging innovations like biosensor-integrated smart implants and nanotechnology are still investigational, offering potential for real-time monitoring and early intervention in the future.

Despite progress, notable limitations remain. Long-term data on newer materials and regenerative solutions are limited, especially in complex or diverse clinical scenarios. AI systems may inherit diagnostic bias from non-representative training data. Biosensor-enabled and gene-modified implants remain experimental, with unresolved concerns about integration, durability, and patient acceptance. High treatment costs, limited access, and regulatory barriers continue to restrict widespread adoption. Ethical concerns surrounding AI-driven decisions, gene-based therapies, and equitable access require careful governance. Moving forward, the integration of sustainable practices, AI, and personalized care can enhance predictability and affordability. Implant dentistry is positioned to reshape oral rehabilitation, but its future success depends on strong evidence, ethical responsibility, and adaptive regulation.
